# Requests for Information

**Published:** 1907-09-21

**Authors:** Conrad W. Thies

**Affiliations:** Secretary, Royal Free Hospital, London


### The Hospital Secretary's Column

[Contributions to this Column are invited and if accepted will be paid for.]

### REQUESTS FOR INFORMATION.

By CONRAD W. THIES, Secretary, Royal Free Hospital, London.

Every hospital secretary receives frequent requests for information on various subjects connected with his work, and such inquiries are, generally speaking, answered very willingly; some of these applications are, however, both unreasonable and unnecessary, because the information asked for can be readily obtained upon reference to such books as Burdett's "Hospitals and Charities" or other recognised publications on hospital subjects.

I do not think that it is generally known that for some years past the Hospitals' Association has established at 28 Southampton Street, Strand, a most useful reference library of books relating to hospitals and their administration. This library includes the annual reports of all the principal London and provincial hospitals, so that persons seeking for information on hospital subjects can thus readily obtain what they require with little trouble.

During the past few years there has been a considerable increase in the various forms of returns which are required by the three distributing funds in London, and the preparation of these returns absorbs a considerable amount of the time of the secretary and his office staff. I do not suppose that any secretary begrudges the time thus spent, because these returns are necessary, and the statistics and information thus collected serve a very useful purpose. The statistical returns of the prices paid at different hospitals for various articles of consumption which the King's Hospital Fund has collected and published during the past three years, has proved a most valuable source of information to those who are responsible for the administration of our voluntary hospitals, and it has doubtless been the means of effecting some considerable economies.

Now that the three Funds have agreed to a revised uniform system of accounts, it is to be hoped that they will arrange that the annual returns to be made by the hospitals shall also be uniform, as this will save much time in the preparation of the returns.

In order to furnish his committee with full information on special points in hospital administration, it is necessary for the secretary to make requests for such information from other kindred institutions. I venture to suggest that all such inquiries should be made as brief as is practicable, and in every instance such requests should be accompanied by a stamped and addressed envelope for the reply. It is surprising how frequently this is omitted, with the probable result that most of such requests find their way to the waste paper basket.

It has been suggested that great advantage would result from the establishment by the three Funds of a central office in London—somewhat on the lines of the central bureau of the "Assistance Publique" in Paris; which office should collect information and to which all such inquiries might be addressed.

It is evident that there is an entirely mistaken idea in the minds of many persons as to the proper functions and work of a voluntary hospital and its officials; doubtless this misconception is responsible for some of the strange requests which I suppose that every hospital secretary occasionally receives.

As an instance of the kind of inquiry to which I refer, I may mention a letter that I received recently from a Colonial General Agency, which, according to a printed statement on its letter paper, undertakes to furnish its clients with "information on any conceivable matter relating to Africa or Asia":—

The letter which I received from this Agency requested me to inform them what advice our medical staff would give to one of their clients residing in Central Africa, who wrote to them as follows:—

"I cannot walk a hundred yards without being exhausted in breathing—choked up in my chest as if I am going to die, and as soon as I am excited I suffer the same feeling; in climbing upstairs or climbing a hill I feel quite choked, as if I will drop down. I am made to understand by the doctors here that the value of my heart is weak, and that I should stop drinking and smoking—drinking I have stopped, but smoking—I have imbibed so much in it that I tried only to do the less of it. Besides suffering from this I am strictured, etc."

It is difficult to understand upon what grounds a commercial agency should send such an unreasonable request to the secretary of a public institution.

I may add that no suggestion was made by this Agency that they were prepared to pay any fee for the advice thus asked for.

I have before me as I write a very long letter addressed to me from the inmate of one of the large lunatic asylums, in which the writer very intelligently furnishes detailed particulars and plans of an ingenious device for preventing accidents on tramcars, which he begs me to bring to the notice of the London County Council.

Amongst my collection of such letters I have a very lengthy one from a young man in Cornwall, in which he tells me his life-history from his earliest infancy, with detailed particulars of his many ailments, and he requests me to find him employment in the office of a London hospital, for which position he thinks his frequent illnesses have specially qualified him, although his handwriting and orthography leave much to be desired.

Another form of application which I frequently receive in my official capacity are urgent requests from distressed persons for financial assistance, usually asked for in the shape of a temporary loan; judging from some of these requests there is evidently an impression on the part of the writers that hospital secretaries are entrusted by wealthy and charitably disposed persons with unlimited funds to be dispensed by them amongst needy applicants; possibly the existence of the Samaritan Funds in connection with some hospitals is responsible for this misunderstanding.

